# Supplementary material for: Joint sequencing of human and pathogen genomes reveals the genetics of pneumococcal meningitis
Source: Nat Commun. 2019 May 15;10:2176. doi: 10.1038/s41467-019-09976-3 (PMC6520353; doi:10.1038/s41467-019-09976-3)
Supplement: Supplementary file 1 — Supplementary Information [file 41467_2019_9976_MOESM1_ESM.pdf]

Supplementary tables

Supplementary table 1: Clinical charateristics of 1168 patients with community-acquired bacterial meningitis. Data are number/number evaluated (percentage), continuous data are mean ±SD. [b]Systolic blood pressure was evaluated in 1146 patients, heart rate in 1133, temperature in 1153. [c]Glasgow Coma Scale score was evaluated in 1163 patients. [d]CSF opening pressure was evaluated in 439 patients, CSF WBC count in 1102 patients, CSF protein in 1113, CSF blood:glucose ratio in 1086.

Characteristic	Value
Age – (yr)	56 ± 17
Male sex – no (%)	595/1168 (50%)
Duration of symptoms <24 hr	525/1125 (47%)
Pretreatment with antibiotics	139/1138 (12%)
Positive blood cultures	764/1052 (73%)
**Predisposing conditions**	
Otitis or sinusitis	425/1161 (37%)
Pneumonia	94/1127 (8%)
Immunocompromised	291/1168 (25%)
**Symptoms and signs on presentation**[b]	
Headache	884/1047 (84%)
Neck stiffness	827/1109 (76%)
Systolic blood pressure – mmHg	144 ± 28
Heart rate – beats/min	99 ± 21
Body temperature – °C	38.7 ± 1.3
Focal neurologic deficits	309/1164 (26%)
**Score on Glasgow Coma Scale**[c]	11 ± 3
<8 indicating coma	133/1163 (11%)
**Indexes of CSF inflammation**[d]	
Opening pressure (mm H_2O)	38 ± 12
White blood cell count (/mm^3)	6784 ± 21858
White blood cell count <1mm^{-3}	327/1102 (30%)
Protein – g/L	4.4 ± 3.7
CSF: blood glucose ratio	0.16 ± 0.19
**Complications**	
Cardiorespiratory failure	227/1139 (19%)
Focal neurologic deficits	239/1134 (21%)
Seizures	131/1120 (12%)
**Score on Glasgow Outcome Scale**	
1 – death	76 (7%)
2 – vegetative state	2 (0.2%)
3 – severe disability	46 (4%)
4 – moderate disability	197 (17%)
5 – good recovery	845 (72%)

Supplementary table 2: Clinical charateristics of 741 patients with community-acquired pneumococcal meningitis. Data are number/number evaluated (percentage), continuous data are mean ±SD. [b]Systolic blood pressure was evaluated in 727 patients, heart rate in 719, temperature in 732. [c]Glasgow Coma Scale score was evaluated in 738 patients. [d]CSF opening pressure was evaluated in 278 patients, CSF WBC count in 704 patients, CSF protein in 706, CSF blood:glucose ratio in 703.

Characteristic	Value
Age – (yr)	58 ± 14
Male sex – no (%)	358/741 (48%)
Duration of symptoms <24 hr	357/714 (50%)
Pretreatment with antibiotics	84/704 (12%)
Positive blood cultures	534/652 (82%)
**Predisposing conditions**	
Otitis or sinusitis	338/735 (46%)
Pneumonia	70/707 (10%)
Immunocompromised	189/741 (26%)
**Symptoms and signs on presentation**[b]	
Headache	551/656 (84%)
Neck stiffness	538/698 (77%)
Systolic blood pressure – mmHg	148 ± 28
Heart rate – beats/min	100 ± 21
Body temperature – °C	38.8 ± 1.3
Focal neurologic deficits	204/738 (28%)
**Score on Glasgow Coma Scale**[c]	11 ± 3
<8 indicating coma	98/738 (13%)
**Indexes of CSF inflammation**[d]	
Opening pressure (mm H_2O)	41 ± 11
White blood cell count (/mm^3)	6859 ± 23610
White blood cell count <1mm^{-3}	220/704 (31%)
Protein – g/L	4.7 ± 3.5
CSF: blood glucose ratio	0.13 ± 0.17
**Complications**	
Cardiorespiratory failure	168/721 (23%)
Focal neurologic deficits	160/708 (23%)
Seizures	106/716 (17%)
**Score on Glasgow Outcome Scale**	
1 – death	56 (8%)
2 – vegetative state	2 (0.3%)
3 – severe disability	31 (4%)
4 – moderate disability	139 (19%)
5 – good recovery	513 (69%)

Supplementary table 3: Summary of cohorts with available human genotype data. The first section shows cohorts with full genotype data where we performed a GWAS; the second section is cohorts with the summary statistics from an existing GWAS used in meta-analysis only. Sample numbers are after QC.

Cohort	Country	Age	Data	Samples	Phenotype
MeninGene	Netherlands	Adults	Illumina Omni array	1 149	Meningitis
				732	Pneumococcal meningitis
				277	Unfavourable outcome
ALS & BPROOF	Netherlands	Adults	Illumina Omni array	4 836	Controls
Benfield	Denmark	Children	Illumina Omni array	353	Pneumococcal meningitis
				873	Pneumococcal bacteremia
				473	Controls
GOYA	Denmark	Young adults	Illumina quad array	2 805	Controls
UK Biobank	UK	Adults	Affymetrix	843	Meningitis
				336 928	Controls
23andme	European	All	Summary statistics	842	Bacterial meningitis
				82 778	Controls
GenOSept	European	Adults	Summary statistics	220	Pneumococcal bacteremia
WTCCC	UK	Adults	Summary statistics	2 244	Controls

Supplementary table 4: Common variation associated with invasiveness using FaST-LMM. Genes are annotated if the significant locus overlaps it, and intergenic variants are annotated with the nearest downstream genes as noted. Gene ID is the name in the ATCC 700669 reference if present; "core" refers to whether this gene was in the core genome; method describes the type of variant that was found to be associated. No antigen alleles or CNVs were found to be associated.

Gene ID	Annotation	Core	Method	p-value
FM211187.6011	*tlyC*; Membrane protein (upstream)	Yes	Mapped variants	7.7×10^{-31}
FM211187.977	*pbpX*; penicillin binding protein	Yes	Mapped variants	3.6×10^{-18}
FM211187.313	hypothetical protein (upstream)	Yes	Mapped variants	2×10^{-16}
FM211187.1802	*yhfE*; Aminopeptidase (upstream)	Yes	Mapped variants	1.0×10^{-9}
FM211187.1019	*wzh*; capsule synthesis	No	Mapped variants	3.6×10^{-9}
FM211187.150	*comA*; bacteriocin/competence (upstream)	Yes	Mapped variants	9.9×10^{-9}
FM211187.3083	*pbl3e*/*pldT*; bacteroicin	No	COG absent	4.0×10^{-10}
N/A	transcriptional regulator (pseudogene)	No	COG absent	1.4×10^{-8}
FM211187.3090	bacteriocin precursor	No	COG absent	1.7×10^{-8}
FM211187.6181	FtsX-family transport protein (ABC transporter permease)	No	COG alleles	4.7×10^{-9}
FM211187.6189	C4-dicarboxylate (citrate) ABC transporter	Yes	COG alleles	1.4×10^{-7}
FM211187.5843	23S rRNA (uracil-5-)-methyltransferase RumA2	Yes	COG alleles	5.5×10^{-7}
FM211187.939	galactose-6-phosphate isomerase	No	K-mers	3.0×10^{-60}
N/A	phage-related chromosomal island protein	No	K-mers	3.0×10^{-60}
FM211187.4259	Peptidase U32	Yes	K-mers	1.7×10^{-59}
FM211187.4090	*aroK*; Shikimate kinase	Yes	K-mers	1.7×10^{-59}
FM211187.1923	*yehU*; Sensor kinase	Yes	K-mers	3.1×10^{-59}
FM211187.6369	*patA*; efflux pump (upstream)	Yes	K-mers	2.0×10^{-54}
FM211187.6823	*tauA*; Nitrate/sulfonate/-taurine ABC transporter solute-binding protein	Yes	K-mers	1.9×10^{-43}
FM211187.213	Galactose uptake PTS transporter, IIB subunit	Yes	K-mers	2.5×10^{-42}
FM211187.3677	*pyrB*; Aspartate carbamoyltransferase PyrB	Yes	K-mers	4.6×10^{-38}
FM211187.6594	*ulaA*; Pentose PTS transporter IIA	Yes	K-mers	3.7×10^{-25}

Supplementary table 5: Coding sequences with extreme values of Tajima's *D*, with a difference between carriage and invasive isolates as determined by permutation testing.

Gene ID	Annotation	Invasive *D*	Carriage *D*	Direction
FM211187.1040	*wzx*; capsule synthesis	-2.53094	-1.79867	Negative in invasive
FM211187.5843	23S rRNA (uracil-5-)-methyltransferase RumA2	-2.4028	-1.63478	Negative in invasive
FM211187.2360	*ezrA*; septation ring formation regulator	-1.1051	-2.17726	Negative in carriage
FM211187.4024	replication initiator protein (on ICE)	-1.55767	-2.16733	Negative in carriage
FM211187.4026	hypothetical, contains FtsK gamma domain (on ICE)	-1.61993	-2.21525	Negative in carriage
FM211187.357	bacteriocin	4.19212	1.30796	Positive in invasive
FM211187.420	*tsaB*; tRNA threonylcarbamoyladenosine biosynthesis protein	3.49345	1.39805	Positive in invasive
FM211187.769	aceytltransferase	2.9055	1.80787	Positive in invasive
FM211187.1019	*wzh*; capsule synthesis	2.76882	1.63677	Positive in invasive
FM211187.1802	*yhfE*; Aminopeptidase	2.28654	1.19784	Positive in invasive
FM211187.1804	bacteroiocin	1.94491	-0.56384	Positive in invasive
FM211187.1806	*dacC*; D-alanyl-D-alanine carboxypeptidase	2.23028	0.722447	Positive in invasive
FM211187.5184	*dnaI*; primosomal protein	2.32212	0.197632	Positive in invasive
FM211187.3651	*tarI*; Ribitol-5-phosphate cytidylyltransferase	-0.146237	2.34171	Positive in carriage
FM211187.3804	*nanB*; neuraminidase	1.6805	3.19937	Positive in carriage
FM211187.5053	membrane protein	0.311619	2.46774	Positive in carriage
FM211187.5358	*secY*; accessory secretion system translocase	0.471641	2.36541	Positive in carriage

Supplementary table 6: Burden testing of rare LoF and damaging variants in coding sequences associated with invasive or carriage isolates. P-values are corrected for multiple testing using a Bonferroni correction with the total number of genes.

Gene ID	Annotation	p-value	Class	Direction
FM211187.1036	*wchV*; capsule synthesis	0.0022	LoF	Carriage
FM211187.1143	membrane protein	0.0022	LoF	Carriage
FM211187.1634	*bglG*; transcription antiterminator	0.0022	LoF	Carriage
FM211187.3315	*zmpD*; zinc metalloprotease	0.0022	LoF	Carriage
FM211187.4588	*pclA*; collagen-like surface-anchored protein	0.0022	LoF	Carriage
FM211187.4679	platelet binding phage protein	0.0022	LoF	Carriage
FM211187.4714	prophage protein	0.0022	LoF	Carriage
FM211187.4939	membrane protein	0.0022	LoF	Carriage
FM211187.5113	*nanA*; neuraminidase	0.0022	LoF	Carriage
FM211187.5328	uncharacterised repeat protein	0.0022	LoF	Carriage
FM211187.5369	PsrP glycosyltransferase	0.0045	LoF	Carriage
FM211187.6773	*dusB*; tRNA-dihydrouridine synthase	0.0045	LoF	Carriage
FM211187.1025	*wze*; capsule synthesis	0.0067	LoF	Carriage
FM211187.4017	hypothetical protein (on ICE)	0.0067	LoF	Carriage
FM211187.1040	*wzx*; capsule synthesis	0.0089	LoF	Carriage
FM211187.92	cell wall-binding amidase/autolysin (pseudogene)	0.0089	LoF	Carriage
FM211187.6861	*comFC*; competence	0.011	LoF	Carriage
FM211187.6608	*pcpA*; choline binding protein	0.016	LoF	Carriage
FM211187.4717	prophage protein	0.018	LoF	Carriage
FM211187.2642	chlorohydrolase	0.029	LoF	Carriage
FM211187.5374	PsrP glycosyltransferase	0.038	LoF	Carriage
FM211187.1804	bacteriocin	0.039	LoF	Carriage
FM211187.3950	conjugal transfer protein (on ICE)	0.042	LoF	Carriage
FM211187.3204	*ybaB*; DNA-binding protein	0.0089	Damaging	Carriage
FM211187.4311	multidrug transporter	0.050	Damaging	Carriage
FM211187.4424	sortase-sorted surface anchored protein (pseudogene)	0.0067	LoF	Invasive
FM211187.2661	*bceA*; ABC exporter ATPase	0.0045	Damaging	Invasive
FM211187.3585	*smc*; Chromosome partition protein	0.0045	Damaging	Invasive

FM211187.5524	*trpD*; anthranilate phosphoribosyltransferase	0.0045	Damaging	Invasive
FM211187.2550	*fruA*; Fructose PTS ABC transporter	0.027	Damaging	Invasive
FM211187.3460	*ispA*; Farnesyl diphosphate synthase	0.038	Damaging	Invasive
FM211187.2615	*pfkA*; ATP-dependent 6-phosphofructokinase	0.042	Damaging	Invasive

Supplementary table 7: Antigen classification of *pspA*, *pspC* and *zmpA*. The total number of samples in the genome-to-genome analysis with each allele is shown, and those where an association test performed are noted.

Antigen	Allele	Samples	Tested
pspA	1	214	✓
	2	231	✓
	3	1	-
	4	1	-
cbpA	0	44	✓
	1	6	-
	2	17	-
	3	84	✓
	4	45	✓
	5	60	✓
	6	191	✓
pspC	0	347	✓
	7	7	-
	8	39	✓
	9	45	✓
	10	6	-
	11	3	-
zmpA	1	26	-
	2	236	✓
	3	185	✓

Supplementary table 8: Number of samples in each population cluster. Cluster two is a polyphyletic "bin" cluster. The dominant serotypes for each cluster, where they account for > 50% of the isolates, are listed.

Cluster	Serotype	Samples	Tested
1	4	17	-
2	-	145	✓
3	8/11A/33F	49	✓
4	10A/35F	22	-
5	23A/B/F	32	✓
6	6B	14	-
7	22F	39	✓
8	9N/15B/19A	47	✓
9	3	47	✓
10	7F	55	✓

Supplementary table 9: Comparison of classifiers of antigen alleles. The balanced accuracy is given by the average of $\frac{1}{2}(\text{sensitivity} + \text{specificity})$ for all alleles.

Method	Balanced accuracy
SVM	0.86
kknn	0.73
Random forest	0.50
DAPC	0.14

Supplementary table 10: Factors beta values from bolt-lmm were adjusted by, to convert to ORs for meta-analysis.

Study	Adjusted beta factor	N_{eff}
Dutch meningitis	6.44647	3713.66
Dutch pneumococcal meningitis	8.757922	2543.069
Danish meningitis	4.086244	808.5666
Danish bacteremia	5.524288	2663.148
23andme	NA	3334.086
GenOSept	NA	801.4286
UKB SR meningitis	4.080513	5157.194
UKB SR sepsis	10.47621	1282.525
UKB ICD meningitis	5.839719	2632.319
UKB ICD all	4.110132	5017.843

Supplementary figures

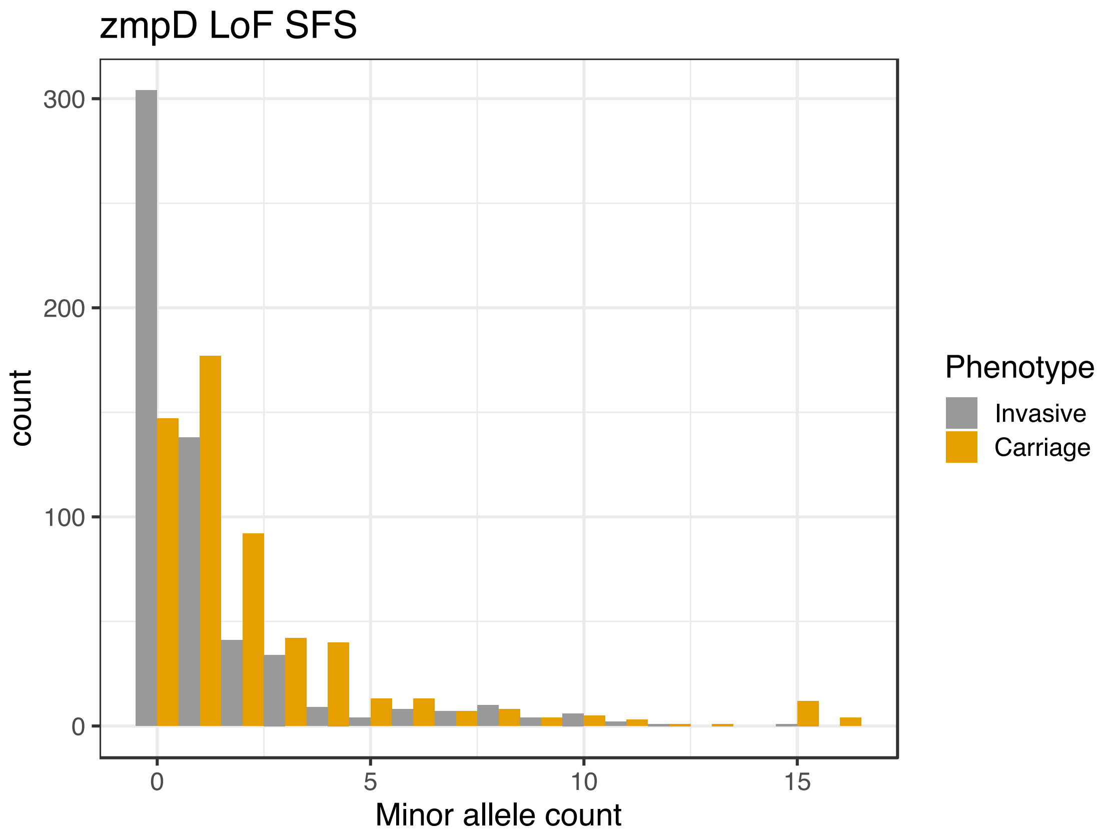

Supplementary figure 1: Site frequency spectrum of rare loss of function variants (MAF $<$ 1%) in *zmpD* in the Dutch cohort. Bars are stratified by phenotype (gray – invasive; orange – carriage).

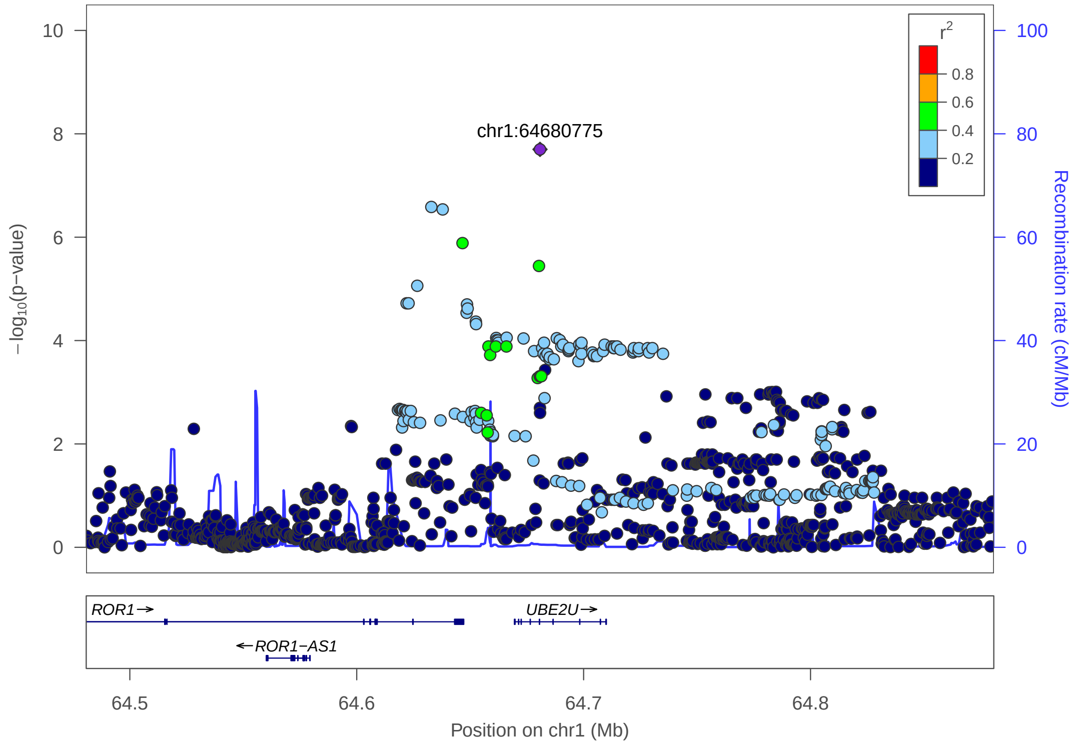

Supplementary figure 2: Locuszoom plot of association on chromosome 1 with unfavourable outcome, which is a zoom of the Manhattan plot on the locus. The lead SNP is a purple diamond, other markers are circles coloured by their r^2 with the lead SNP to show LD. The bottom panel shows annotated genes in the region, with exons as boxes and introns as lines. Recombination rate in cM/Mb is plotted as a pale blue line.

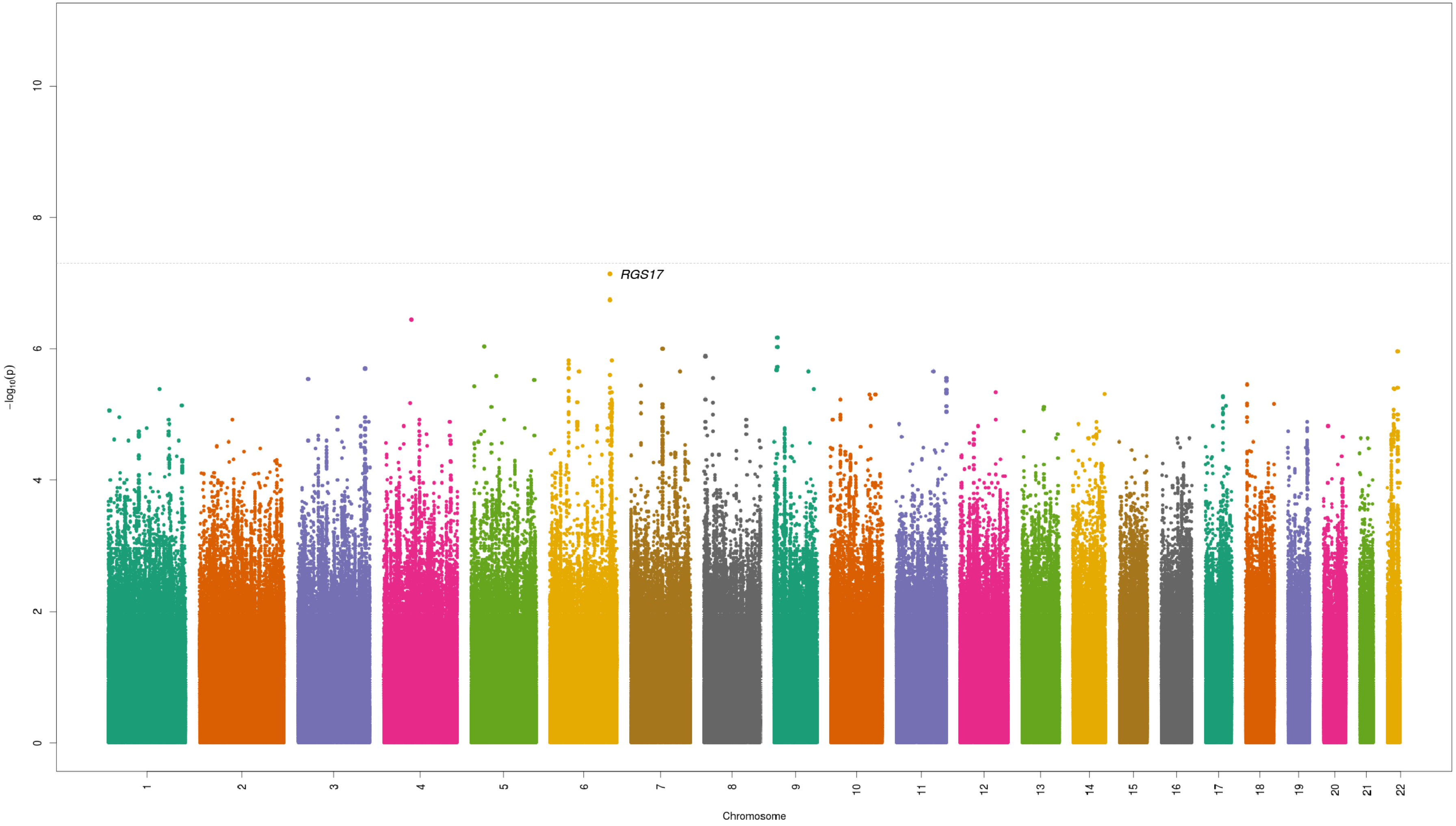

Supplementary figure 3: Manhattan plot from GWAS of all Dutch meningitis cases. Y-axis is $-\log_{10}(p)$ of the association values, x-axis is ordered by marker position on each autosome. Suggestive results are annotated with nearby genes. Genome-wide significance is at 5×10^{-8}.

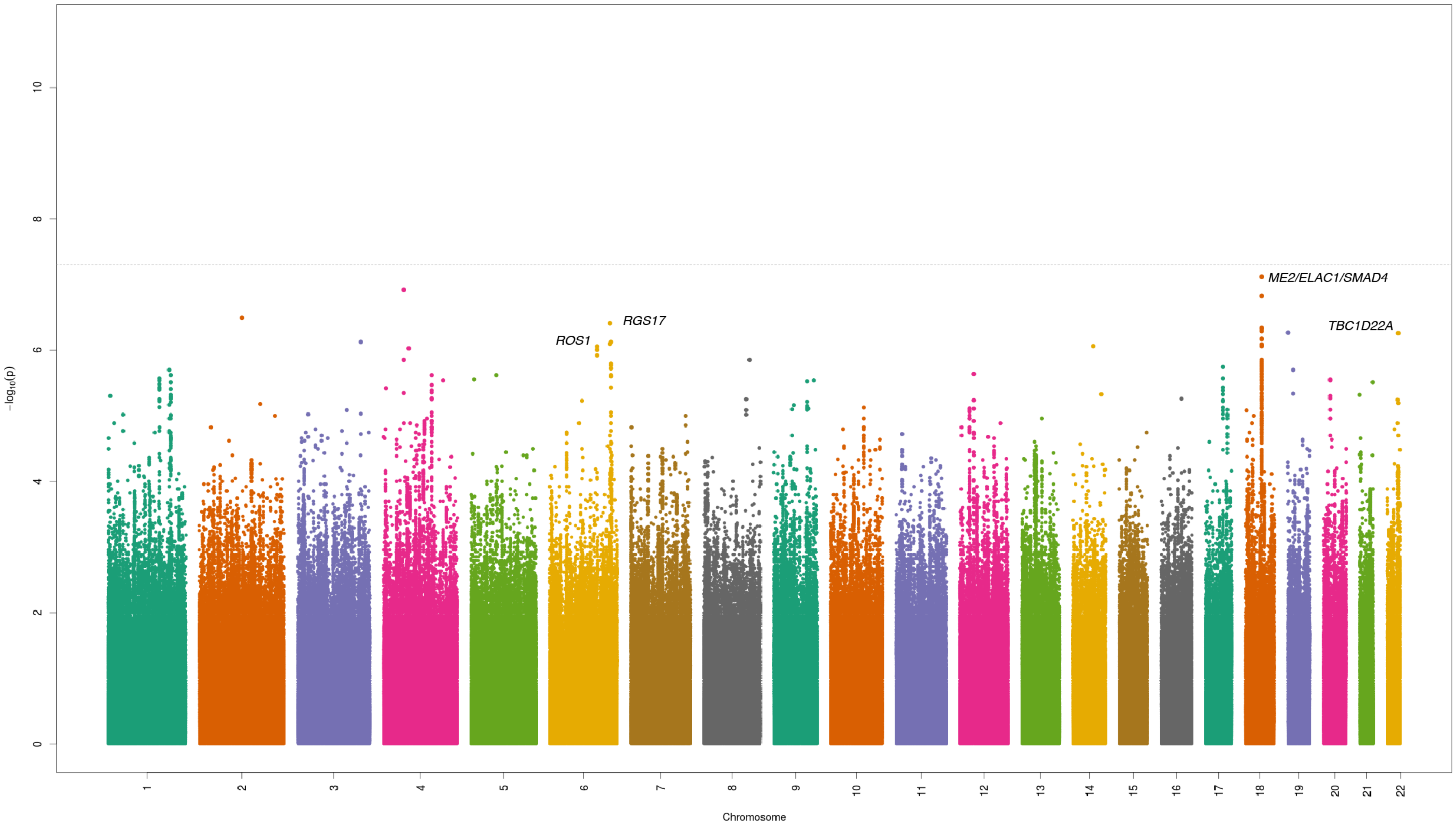

Supplementary figure 4: Manhattan plot from GWAS of Dutch pneumococcal meningitis cases. Y-axis is $-\log_{10}(p)$ of the association values, x-axis is ordered by marker position on each autosome. Suggestive results are annotated with nearby genes. Genome-wide significance is at 5×10^{-8}.

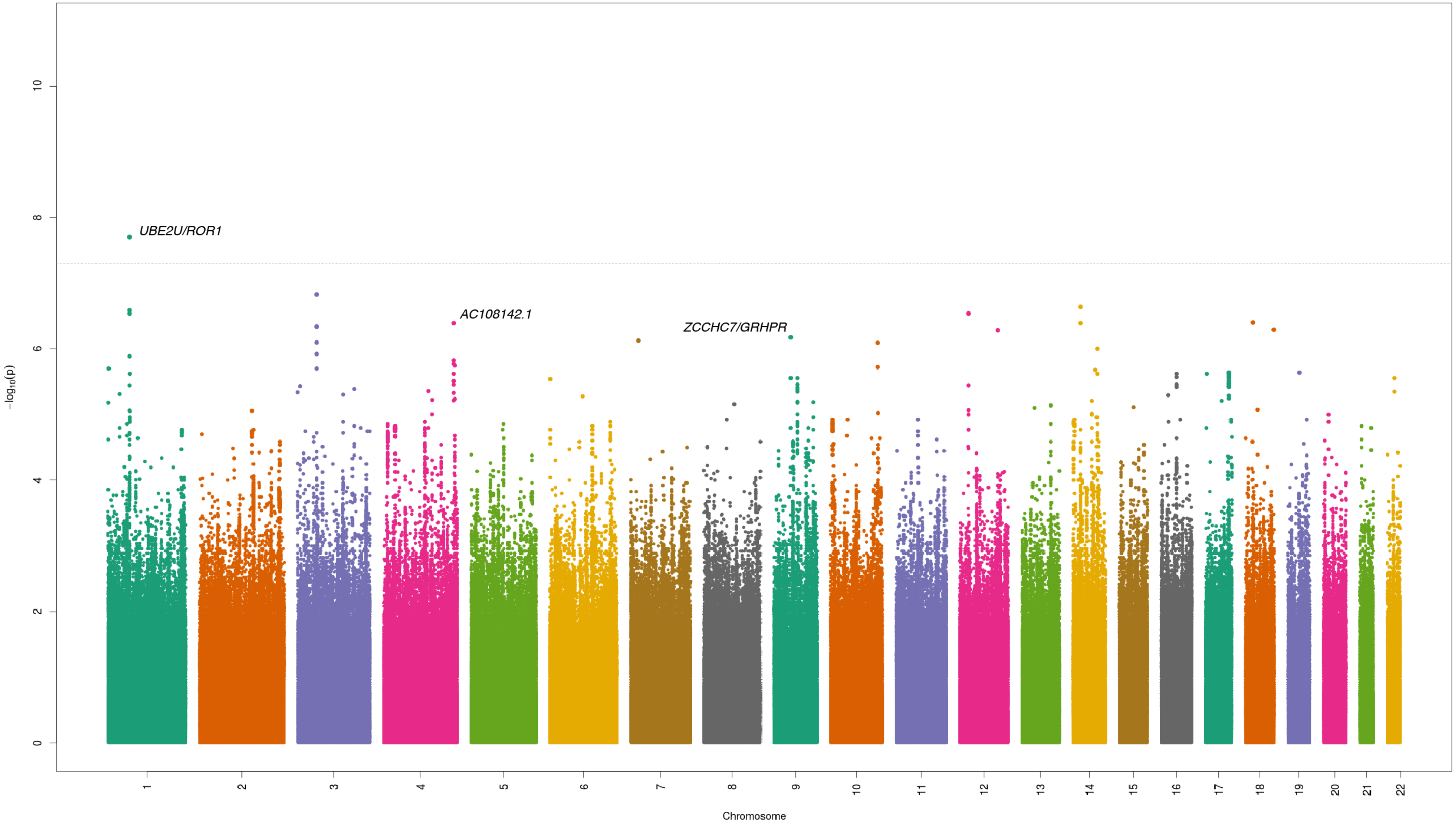

Supplementary figure 5: Manhattan plot from GWAS of all Dutch meningitis cases with an unfavourable outcome. Y-axis is $-\log_{10}(p)$ of the association values, x-axis is ordered by marker position on each autosome. Suggestive results from are annotated with nearby genes. Genome-wide significance is at 5×10^{-8}.

rs12081070 in Macrophages M1 Tissues

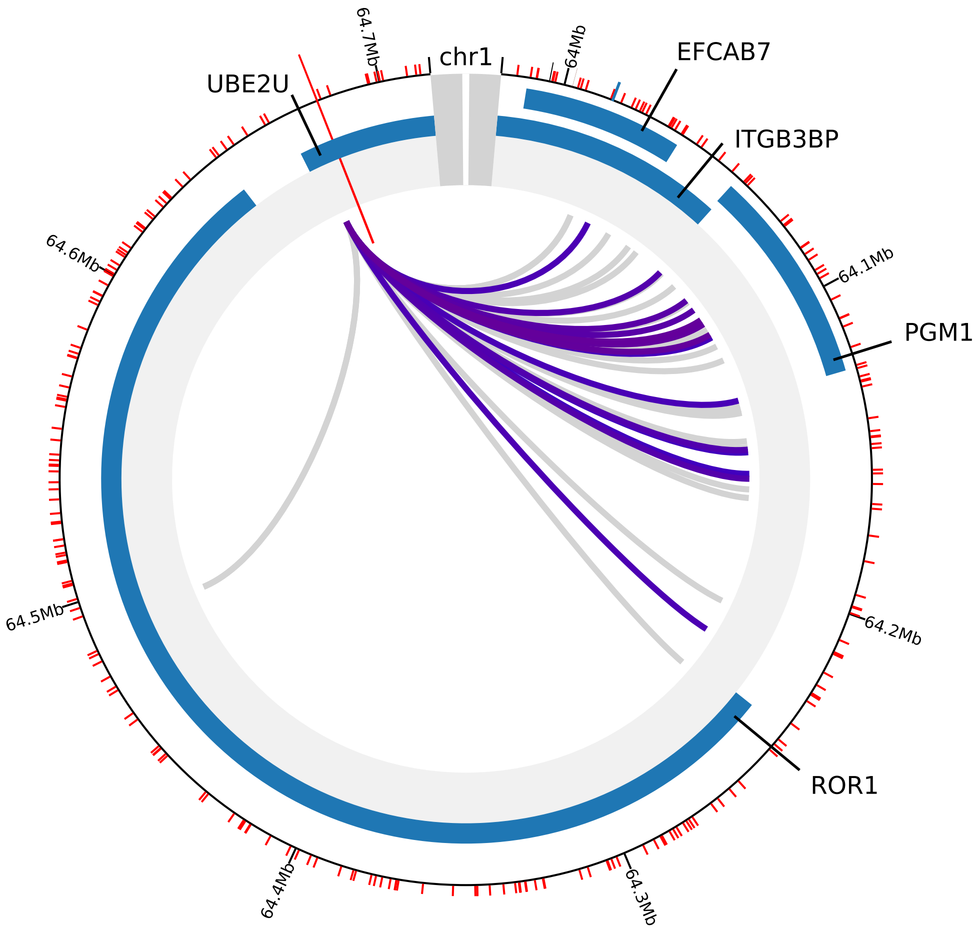

Supplementary figure 6: Neighbouring chromatin interactions with rs12081070 *UBE2U* intronic variant in M1 macrophages. A fragment of chromosome 1 is represented by the outermost track with *HindIII* restriction sites highlighted in red ticks with the rs12081070 variant represented by a long straight red line. Protein coding genes are represented by blue bars and central arcs represent interactions coloured by interaction score (grey <5; purple 5-8; red >8). A number of interactions are observed in macrophages between the variant-containing bait including the *UBE2U* gene and *ROR1* and *PGM1* genes.

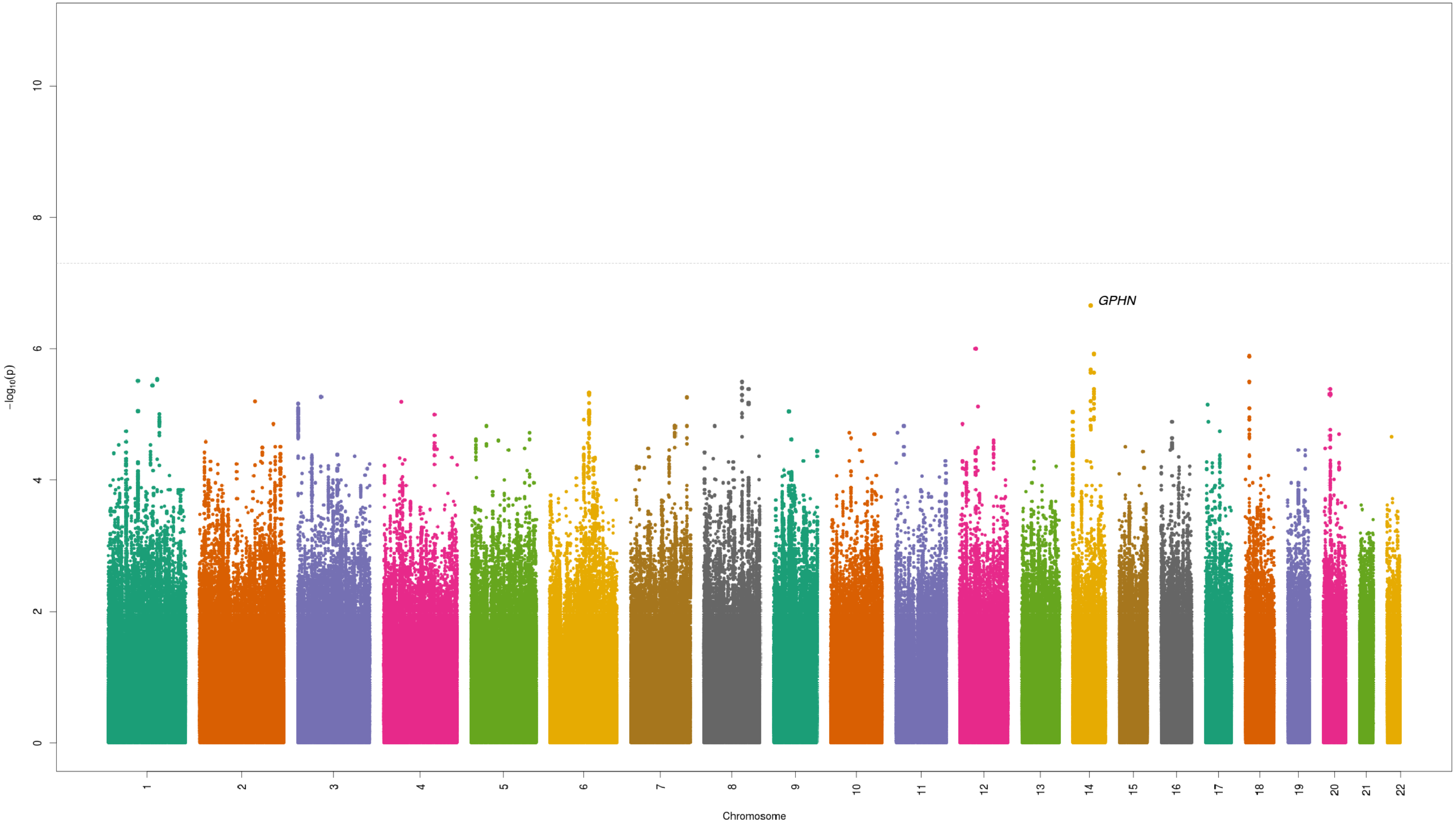

Supplementary figure 7: Manhattan plot from GWAS of Danish pneumococcal meningitis cases. Y-axis is $-\log_{10}(p)$ of the association values, x-axis is ordered by marker position on each autosome. The suggestive results is annotated with nearby genes. Genome-wide significance is at 5×10^{-8}.

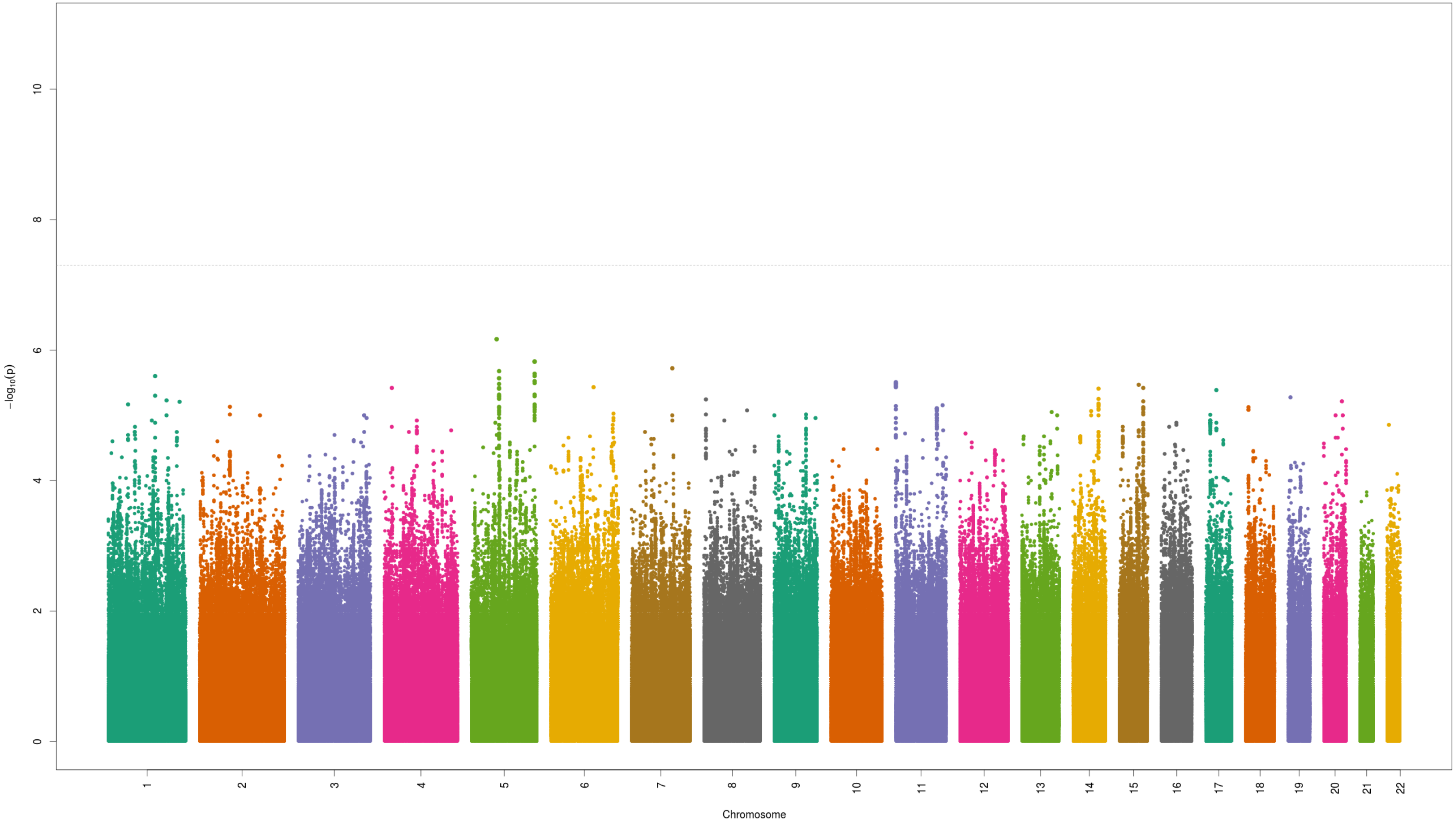

Supplementary figure 8: Manhattan plot from GWAS of all Danish pneumococcal bacteremia cases. Y-axis is $-\log_{10}(p)$ of the association values, x-axis is ordered by marker position on each autosome. Genome-wide significance is at 5×10^{-8}.

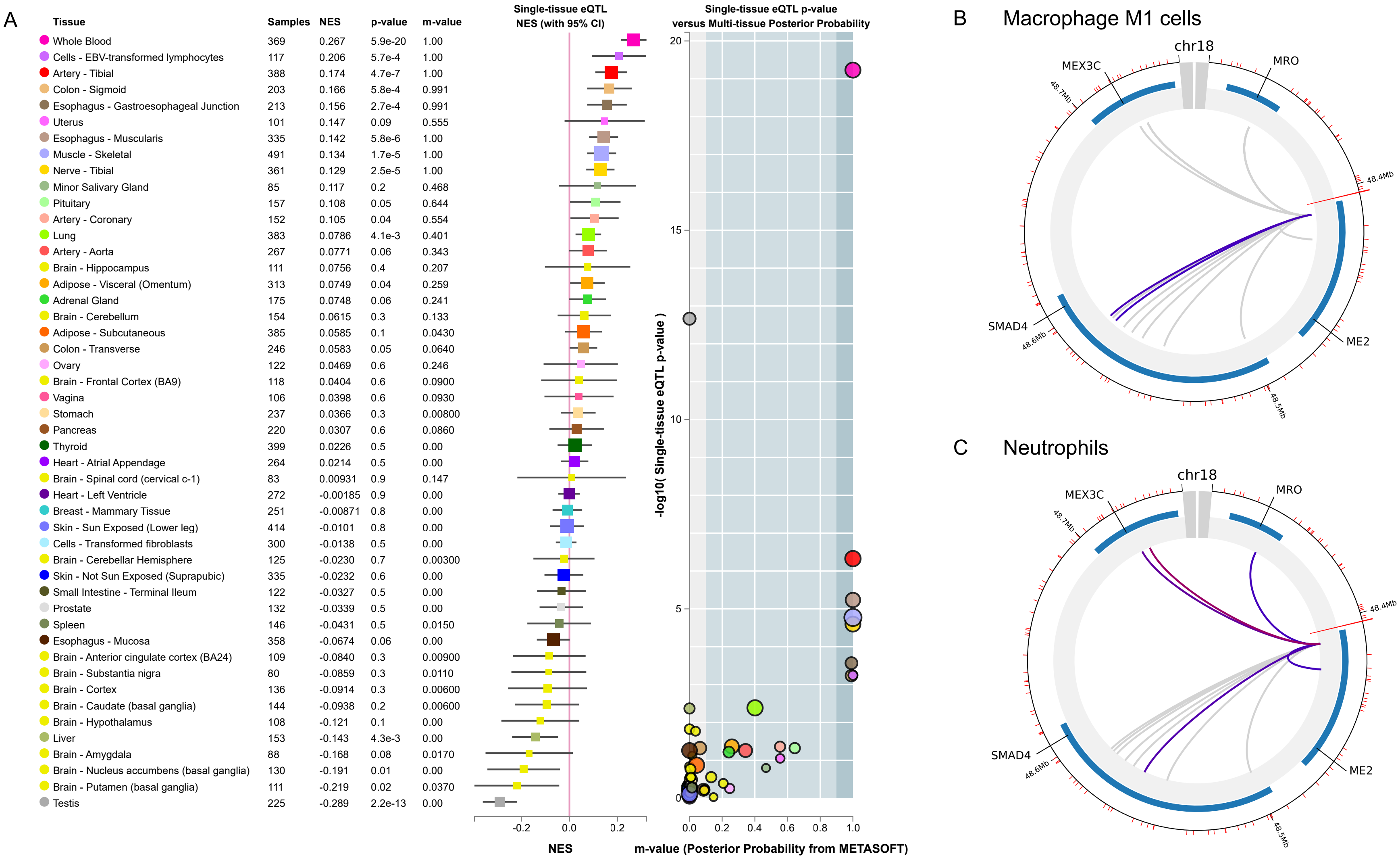

Supplementary figure 9: Multi-tissue eQTL and cell-specific chromatin interactions of rs2850542 *ME2* promoter variant. **A)** eQTL effects of the variant on the same gene in a range of tissues with number of samples tested, normalised effect size (NES) and p and m posterior probability values calculated in METASOFT shown numerically and with $-\log_{10}$ p-values plotted against m-values for each tissue type in the right-hand scatter plot with tissues represented by coloured points sized by sample-size. Chromatin interactions in M1 macrophages **(B)** and neutrophils **(C)** with a fragment of chromosome 18 represented by the outermost track with *HindIII* restriction sites highlighted in red ticks with the rs2850542 variant represented by a long straight red line. Protein coding genes are represented by blue bars and central arcs represent interactions coloured by interaction score (grey <5; purple 5-8; red >8). Consistent interactions are observed between the variant-containing bait including the *ME2* gene and the *SMAD4* gene.

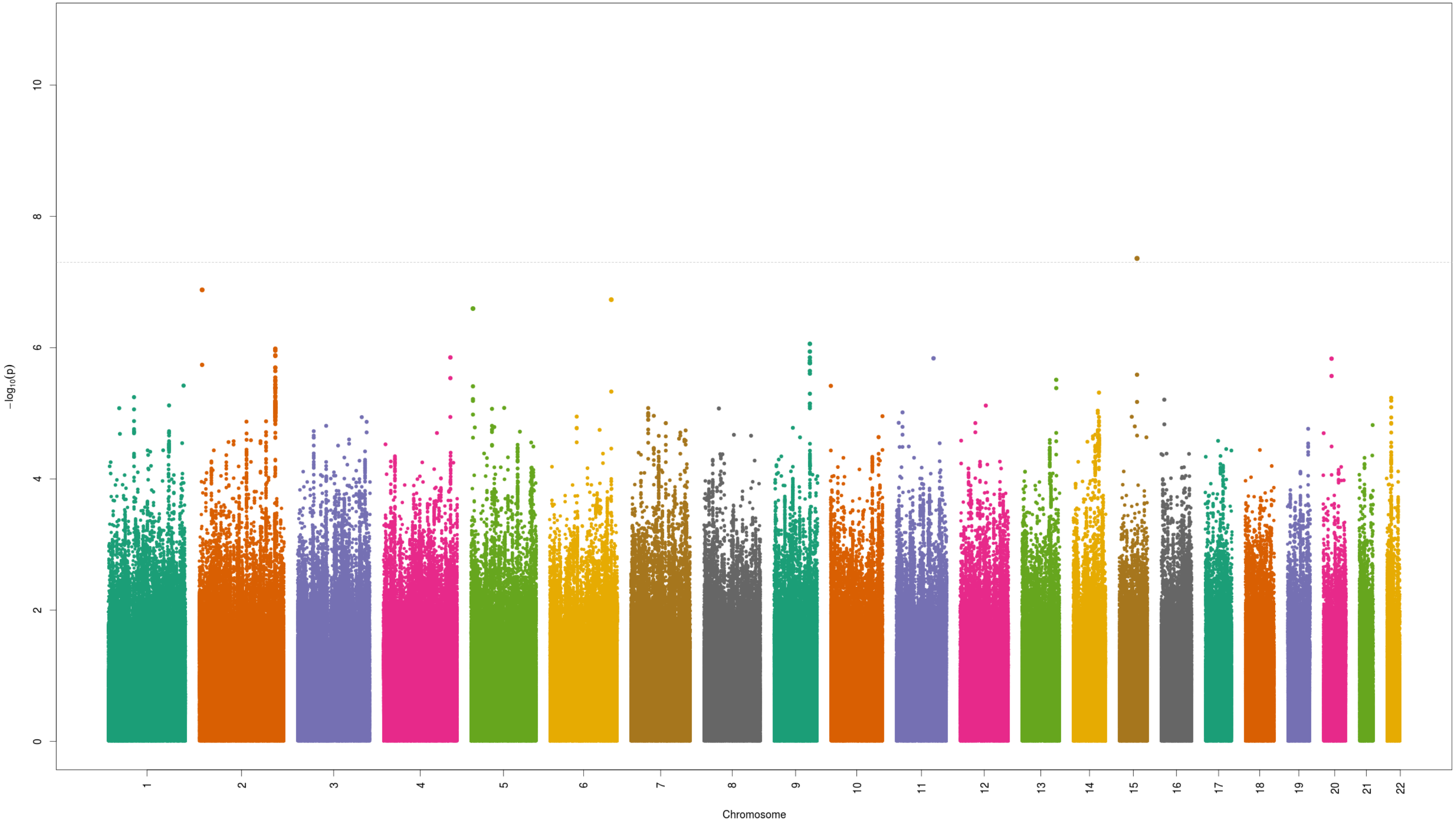

Supplementary figure 10: Manhattan plot from meta-analysis of Meningene, Danish and UK biobank (ICD defined) cohorts. Y-axis is $-\log_{10}(p)$ of the association values, x-axis is ordered by marker position on each autosome. Genome-wide significance is at 5×10^{-8}.

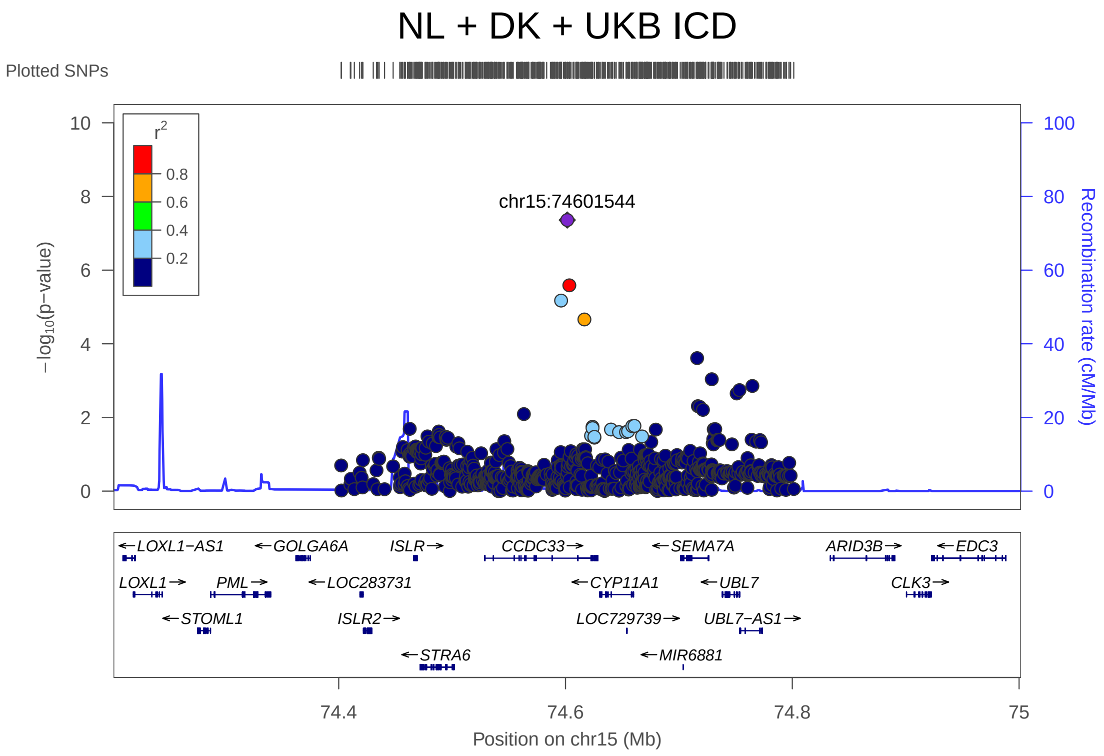

Supplementary figure 11: Locuszoom plot of meta-analysis association on chromosome 15 with meningitis susceptibility, which is a zoom of the Manhattan plot on the locus. The lead SNP is a purple diamond, other markers are circles coloured by their r^2 with the lead SNP to show LD. The bottom panel shows annotated genes in the region, with exons as boxes and introns as lines. Recombination rate in cM/Mb is plotted as a pale blue line.

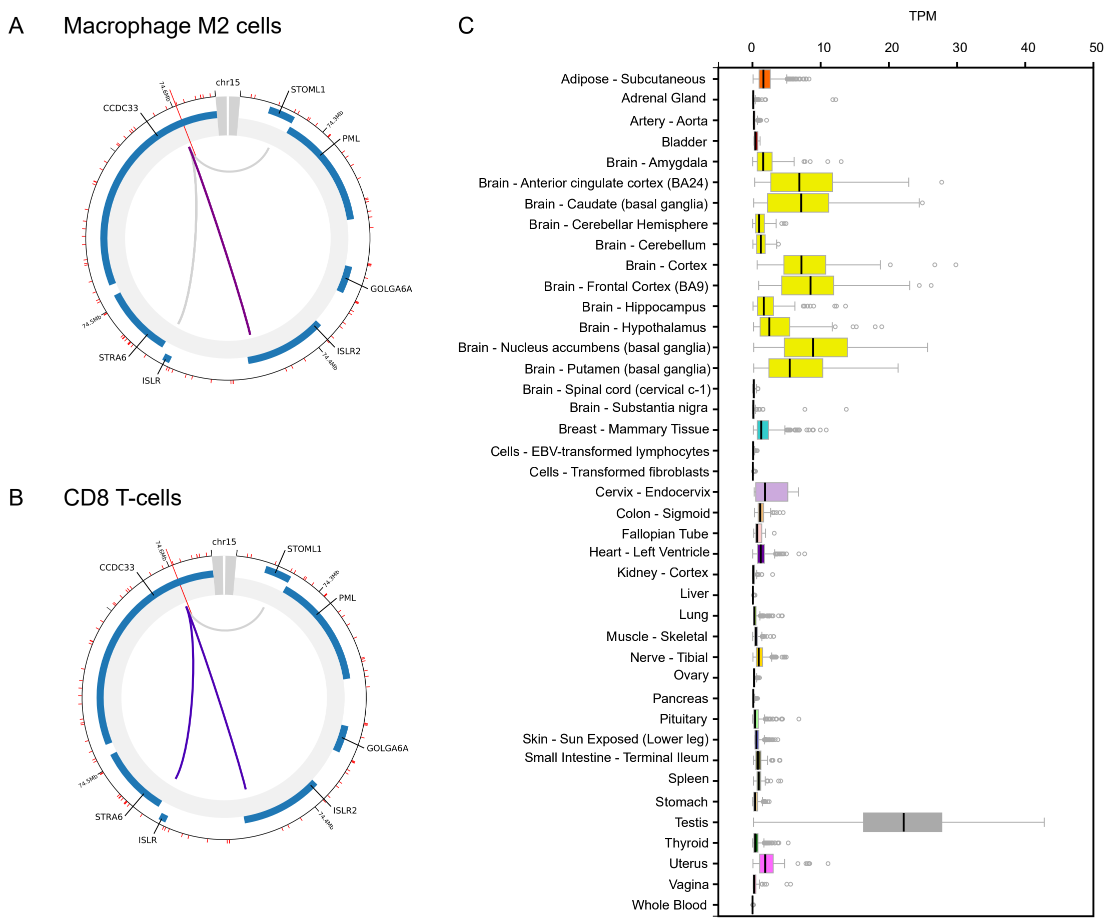

Supplementary figure 12: Cell-specific chromatin interactions of the rs116264669 *CCDC33* intronic variant and tissue-specific *ISLR2* gene expression. Chromatin interactions in M2 macrophages **(A)** and CD8 T-cells **(B)** with a fragment of chromosome 15 represented by the outermost track with *HindIII* restriction sites highlighted in red ticks with the rs116264669 variant represented by a long straight red line. Protein coding genes are represented by blue bars and central arcs represent interactions coloured by interaction score (grey <5; purple 5-8; red >8). Consistent interactions are observed between the variant-containing bait including the *CCDC33* gene and the *ISLR2* gene. **C)** Expression levels of the *ISLR2* gene in multiple tissues coloured by tissue type and measured in transcripts per million (TPM) with significant expression levels in neural tissue and testis.

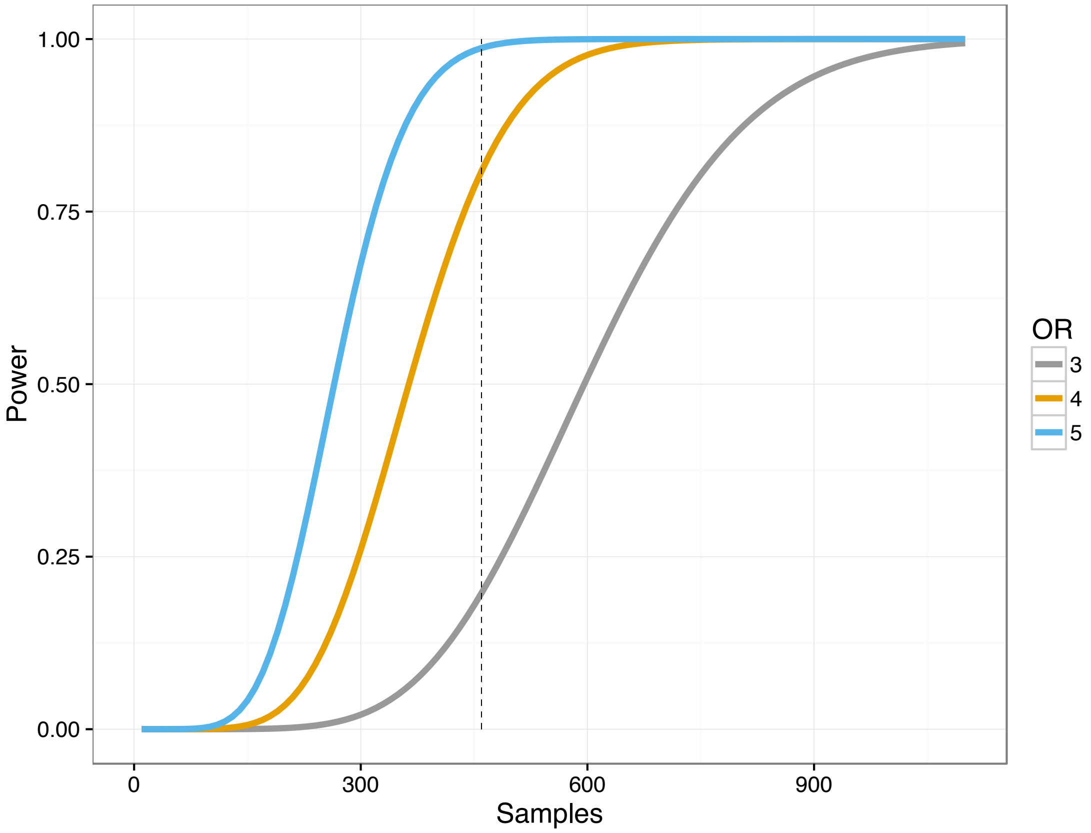

Supplementary figure 13: Power for detecting genome-to-genome interactions. Assuming no population structure effect, the power of detecting an correlation between genome positions at 25 % MAF at a range of ORs. The 460 samples we used in this study is marked as a vertical dashed line.

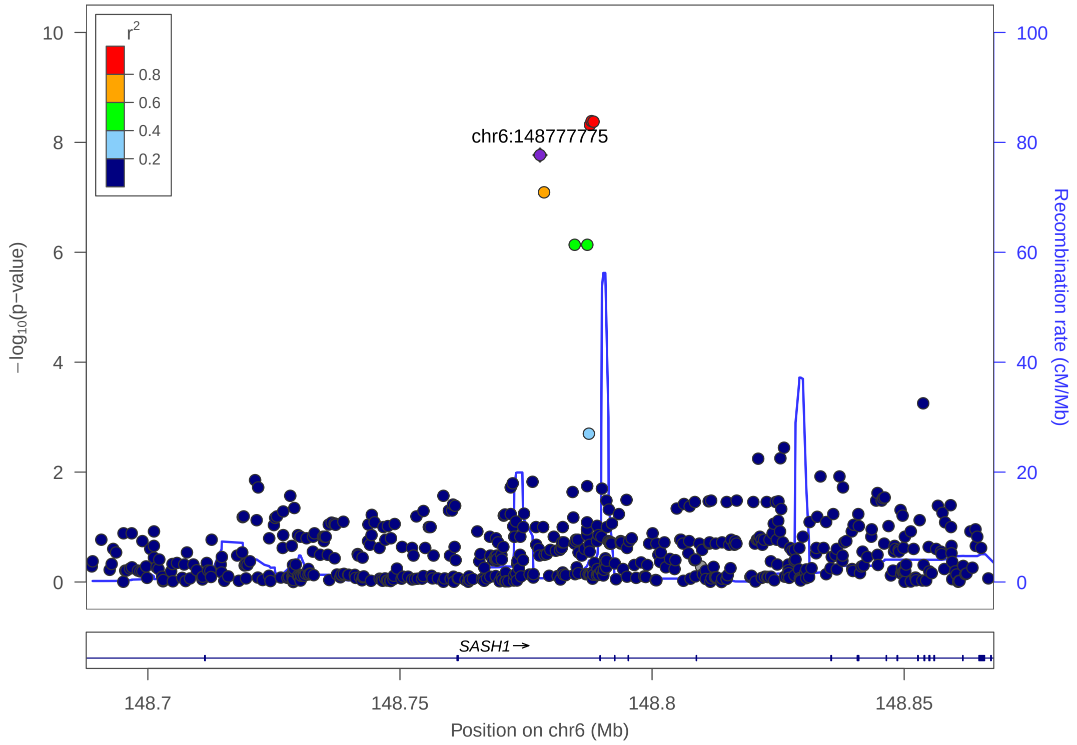

(a) *pspC* allele 8, and chromosome 6 (*SASH1*)

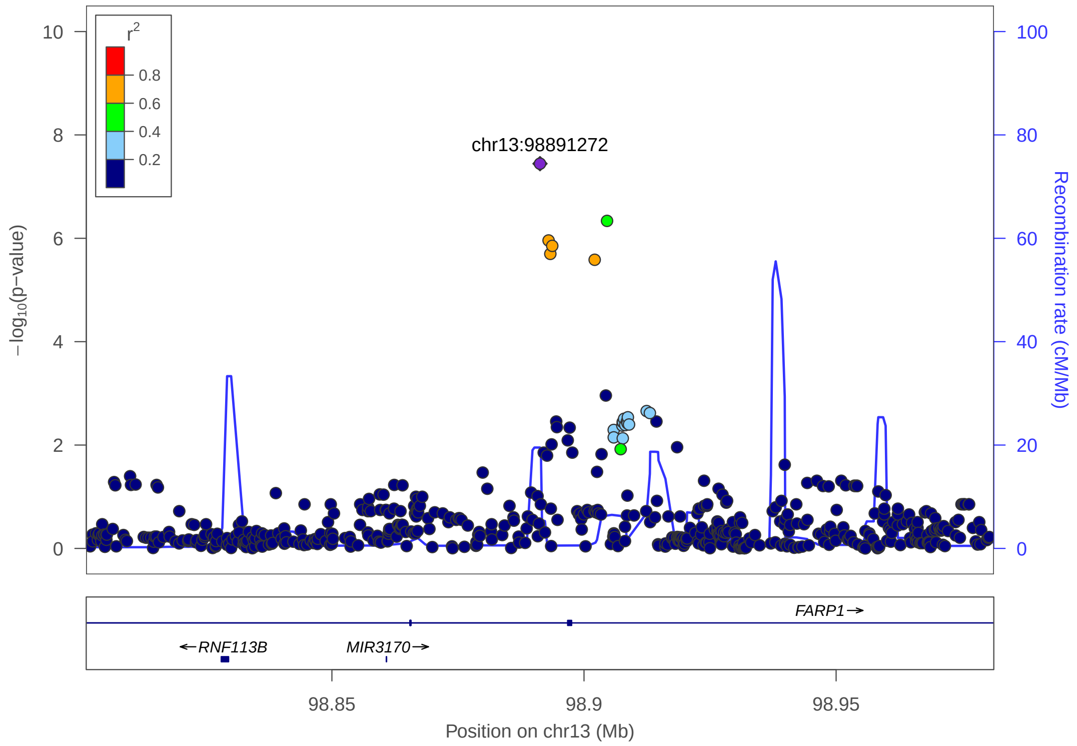

(b) *pspC* allele 9, and chromosome 13 (*FARP1*)

Supplementary figure 14: Locuszoom plot of association between *pspC* allele and imputed human SNPs.

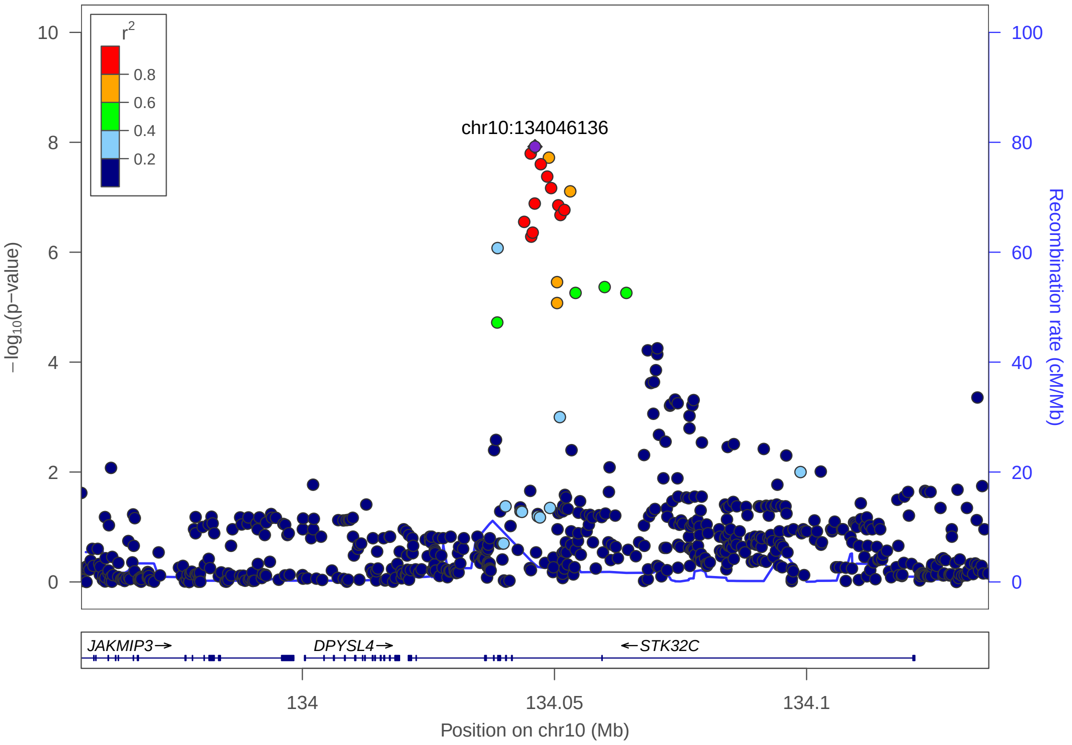

Supplementary figure 15: Locuszoom plot of association on chromosome 10 with sequence cluster 8.

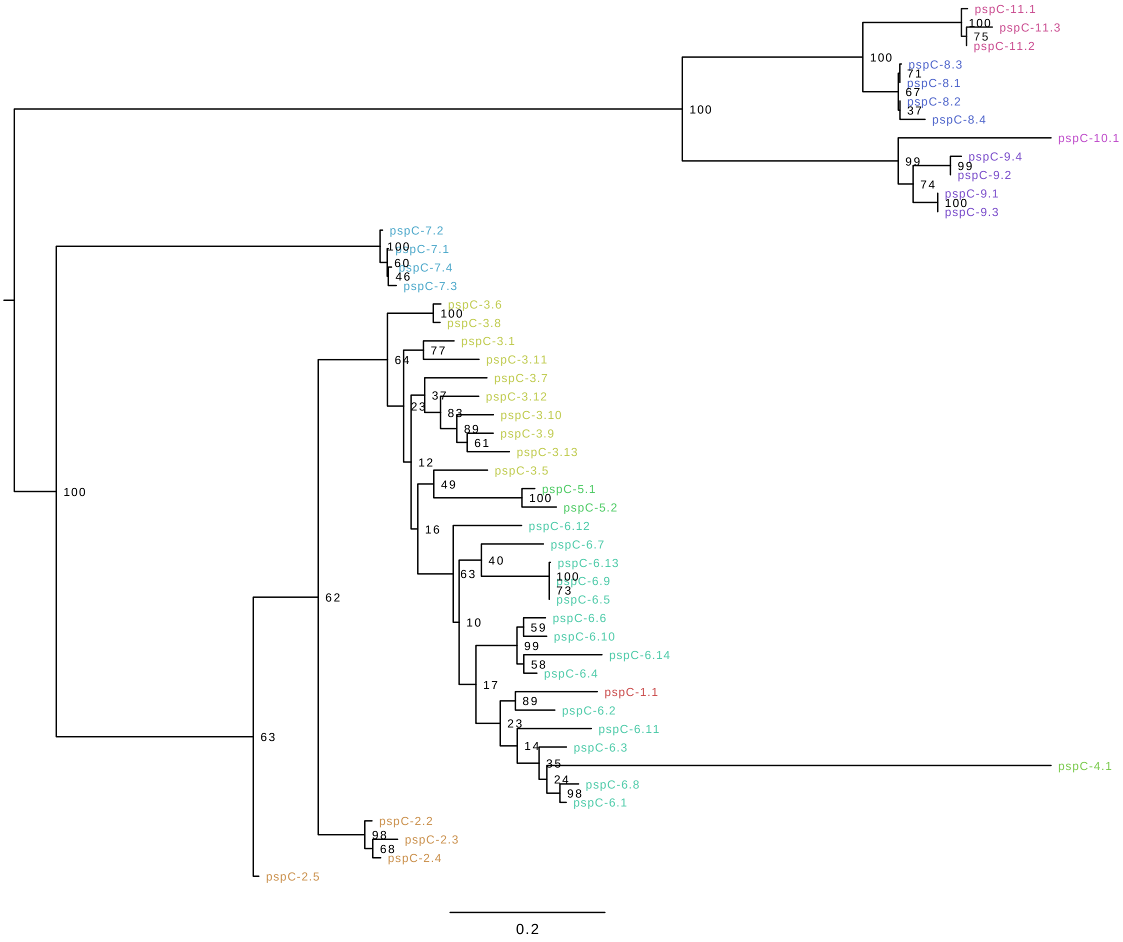

Supplementary figure 16: Maximum likelihood tree of *pspC* protein alignment, with 100 bootstrap replicates (nodes are labelled with bootstrap supports). Tips are coloured by allele group.

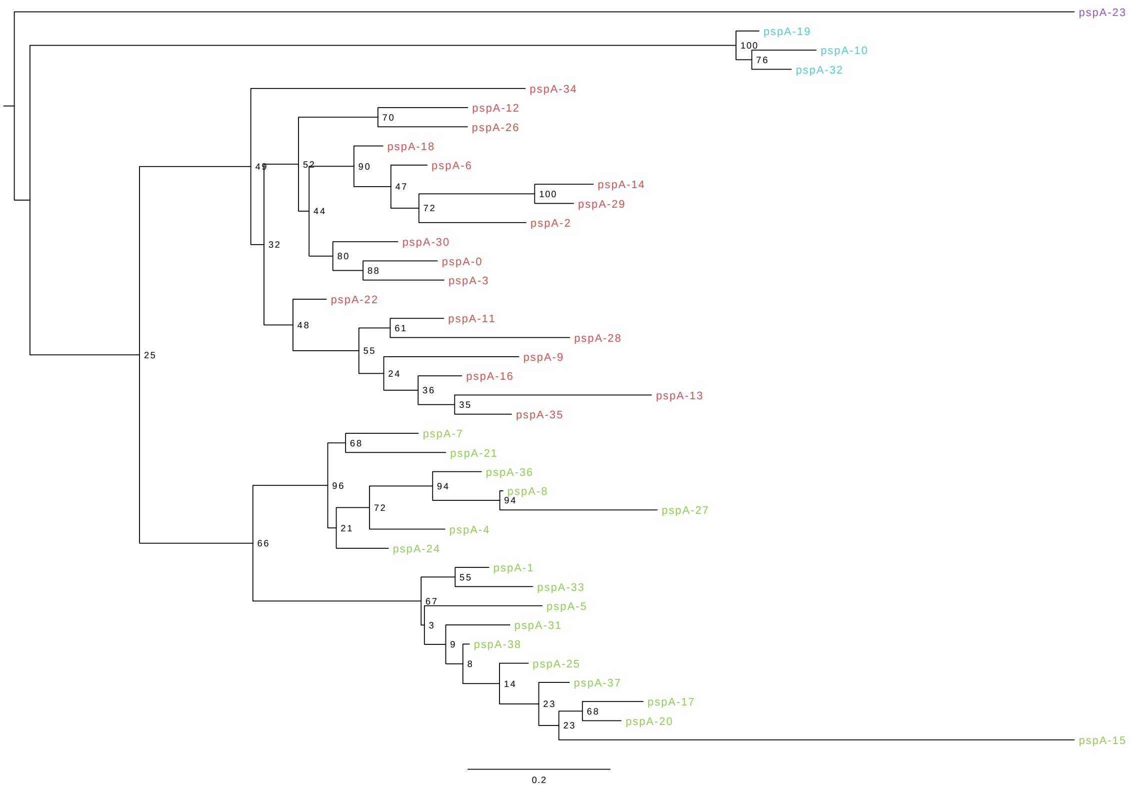

Supplementary figure 17: Maximum likelihood tree of *pspA* protein alignment, with 100 bootstrap replicates (nodes are labelled with bootstrap supports). Tips are coloured by allele group.

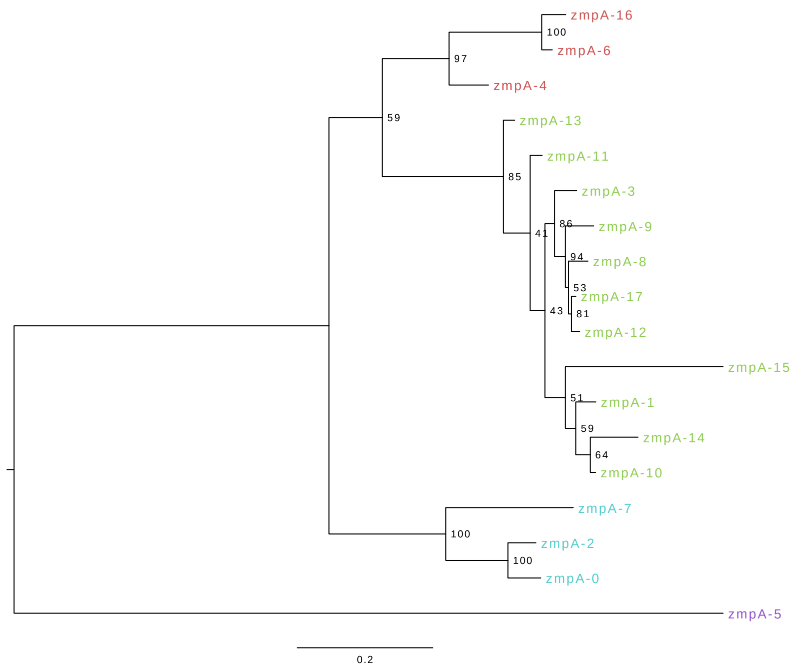

Supplementary figure 18: Maximum likelihood tree of *zmpA* protein alignment, with 100 bootstrap replicates (nodes are labelled with bootstrap supports). Tips are coloured by allele group.

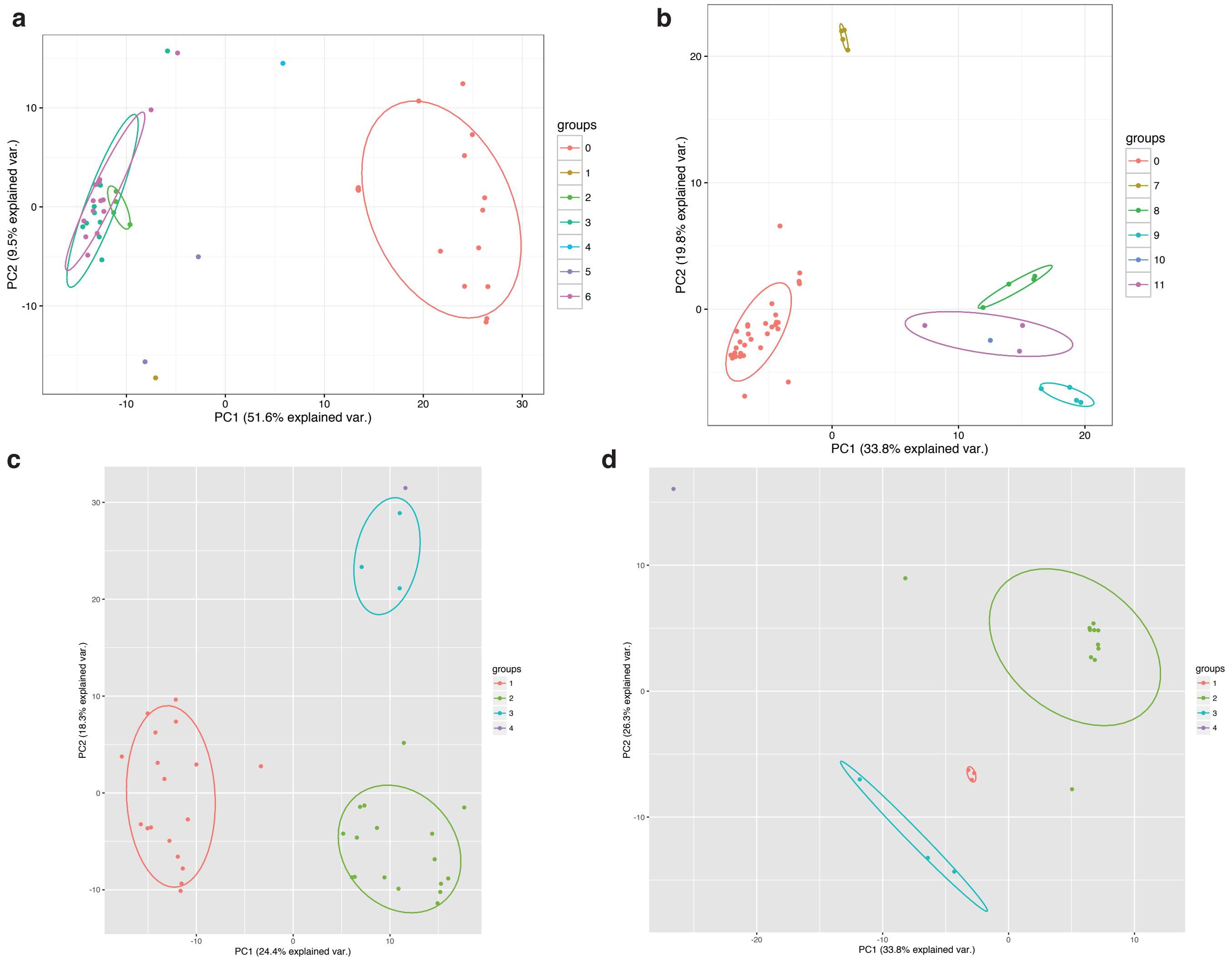

Supplementary figure 19: PCA plots of classifiers used on antigen training data, first two principal components shown in each case. Points are coloured by the allele number, where 0 is a genome without the antigen. Where more than one point is available for a class, an ellipse has been drawn around its centroid. **a)** PspC, alleles 0–6; **b)** PspC, alleles 0,7–11; **c)** PspA, alleles 1–4; **d)** ZmpA, alleles 1–4

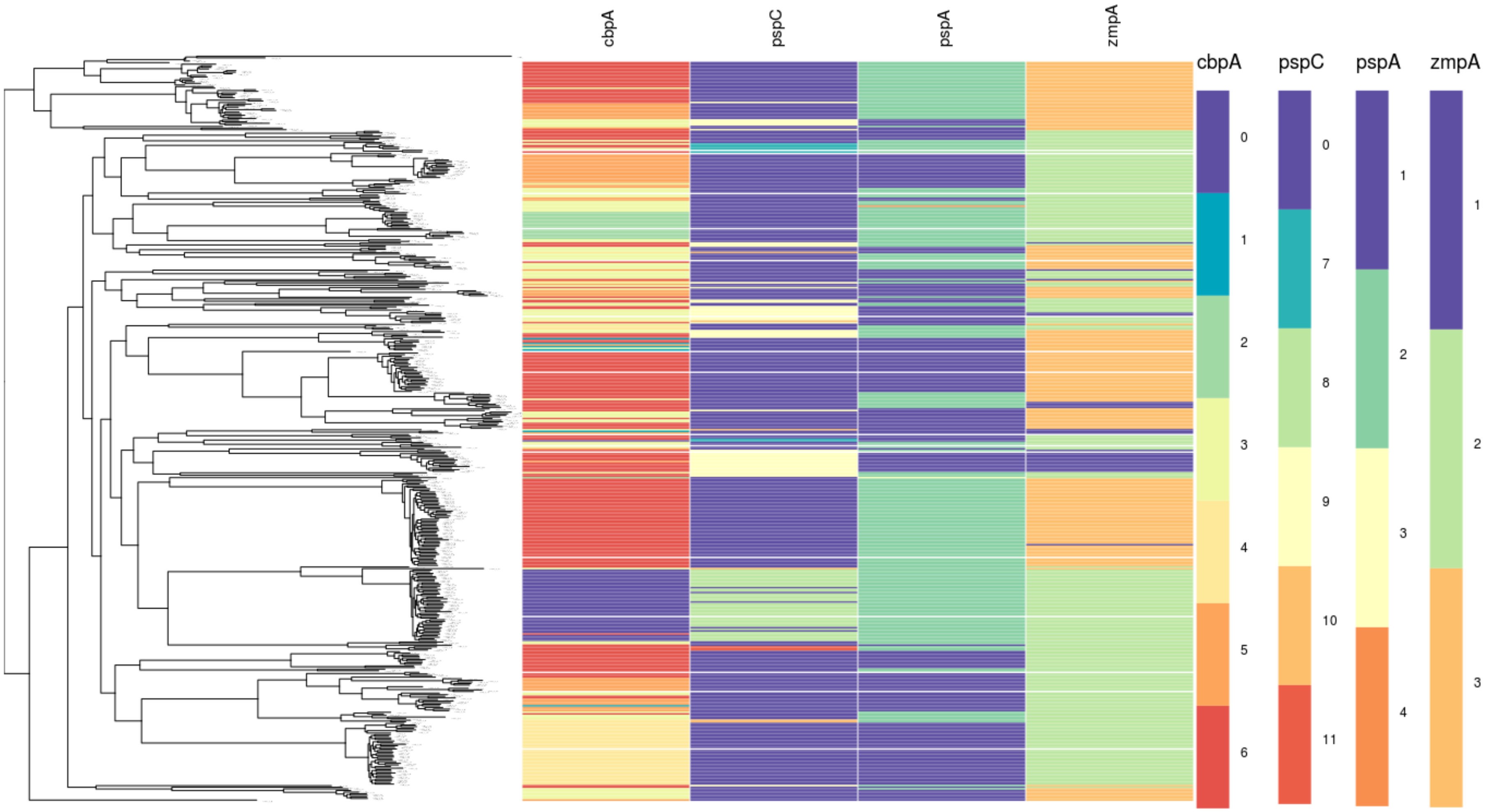

Supplementary figure 20: The inferred allele of pneumococcal antigens *zmpA*, *pspA* and *pspC*. Left: phylogenetic tree of CSF isolates. Right: tips coloured by the inferred allele for three antigens, and key. The first two columns are alleles 1–6 and 7–11 of pspC, which may have two copies present.

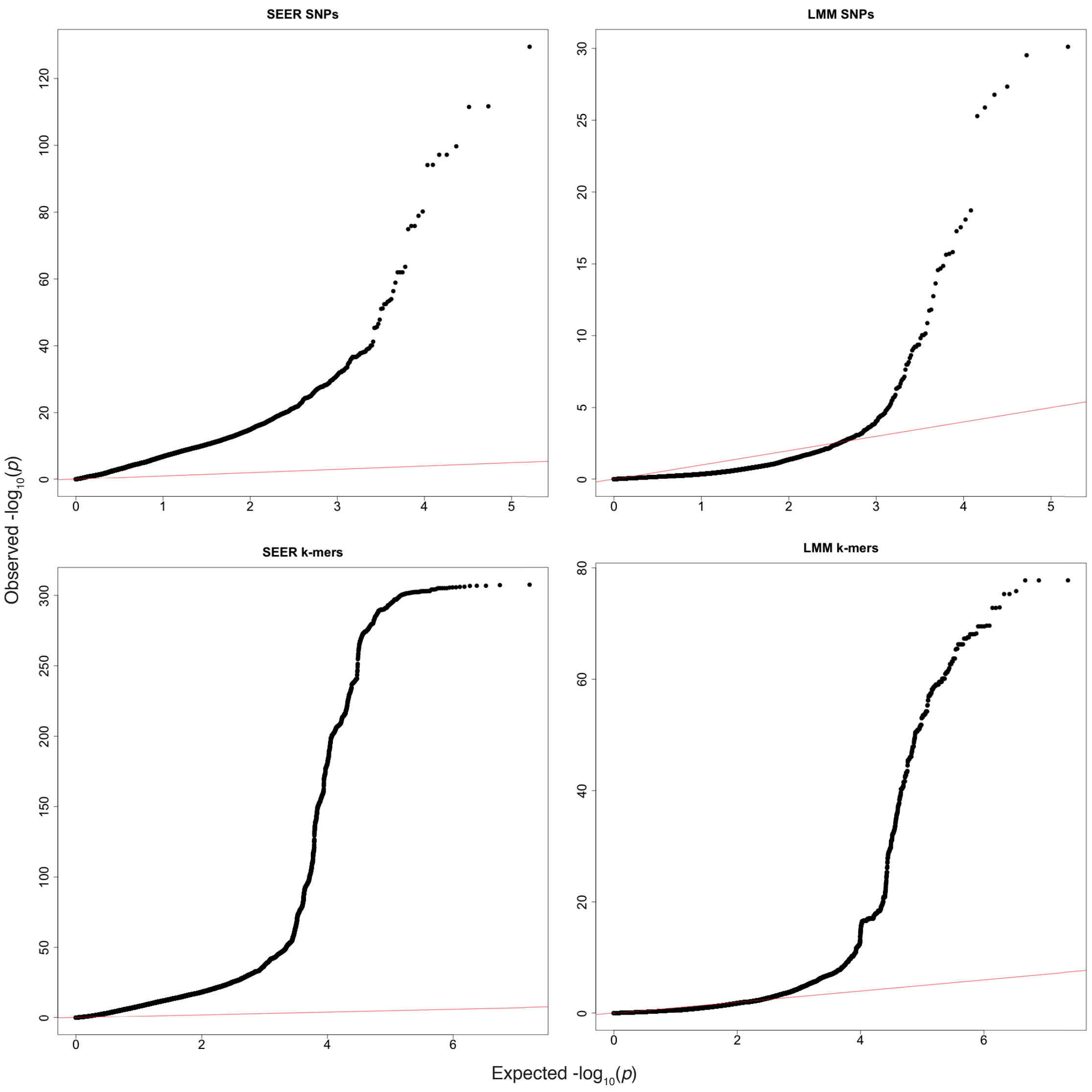

Supplementary figure 21: Quantile-Quantile plots for association of common variation in the pathogen genomes. Red line is for observations following the null-hypothesis of no association, plotted points are observed p-values from each method. Top row: p-values from SNPs and INDELs from mapping; bottom row: p-values from k-mers. Left column: SEER run with the first ten population structure components. Right column: FaST-LMM run on the same input.

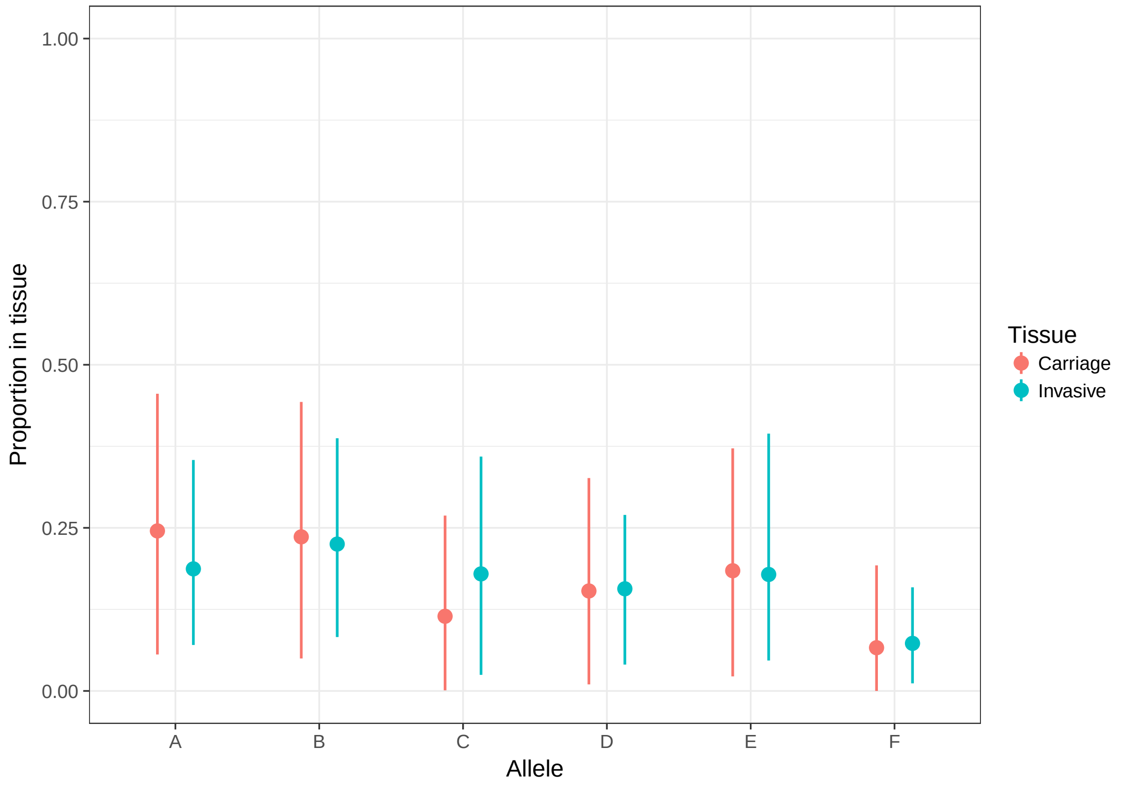

Supplementary figure 22: Mean (points) and 95% highest posterior density (lines) for μ_c in the *ivr* allele model. This shows the proportion of each allele present in each of carriage (red) and meningitis (turquoise) isolates, effectively pooling across all samples.

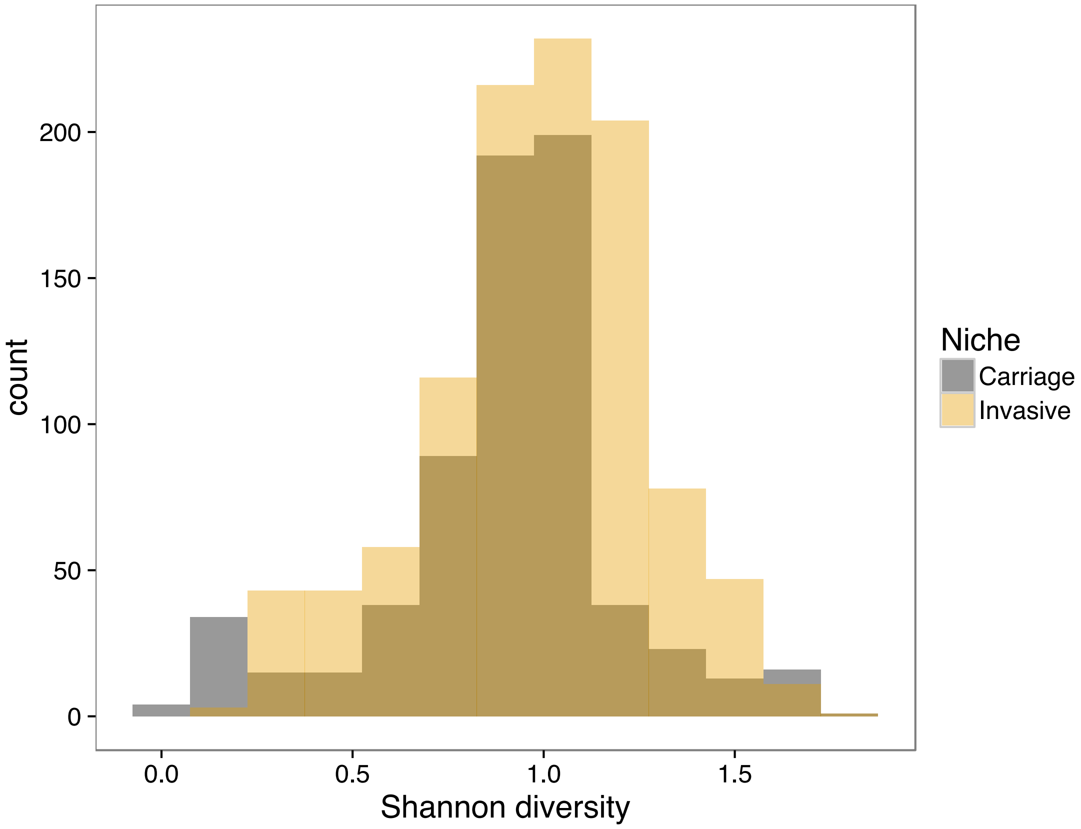

Supplementary figure 23: Histogram of the Shannon diversity of the *ivr* allele makeup in carriage and invasive samples, using the mean frequency of each allele from the highest posterior density.

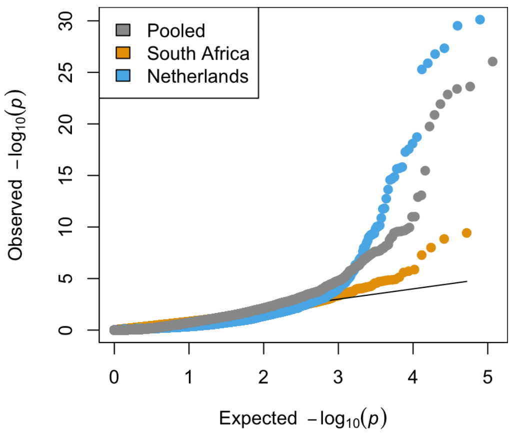

(a) Common SNP and INDEL variants.

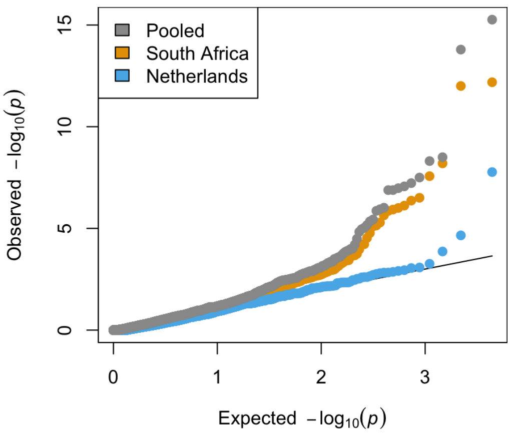

(b) Accessory COG presence/absence.

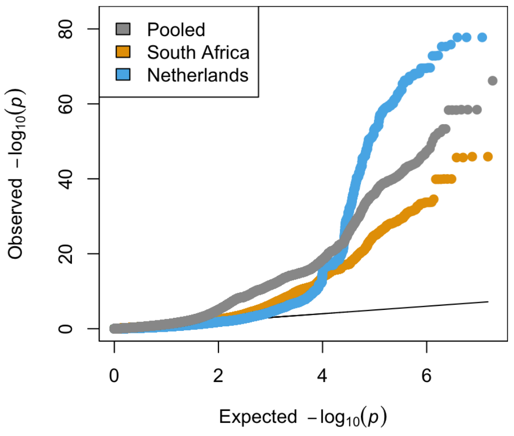

(c) Variable length k-mers.

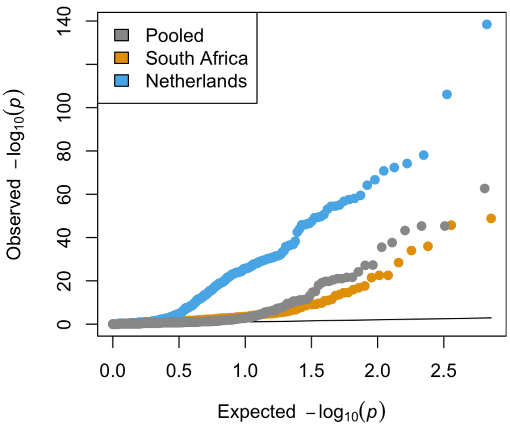

(d) Burden test of rare LoF variants.

Supplementary figure 24: QQ-plots of association of pneumococcal variants. Shown are p-values from both bacterial cohorts individually, and when all the data was pooled. Each panel is for a different category of variant.

a)

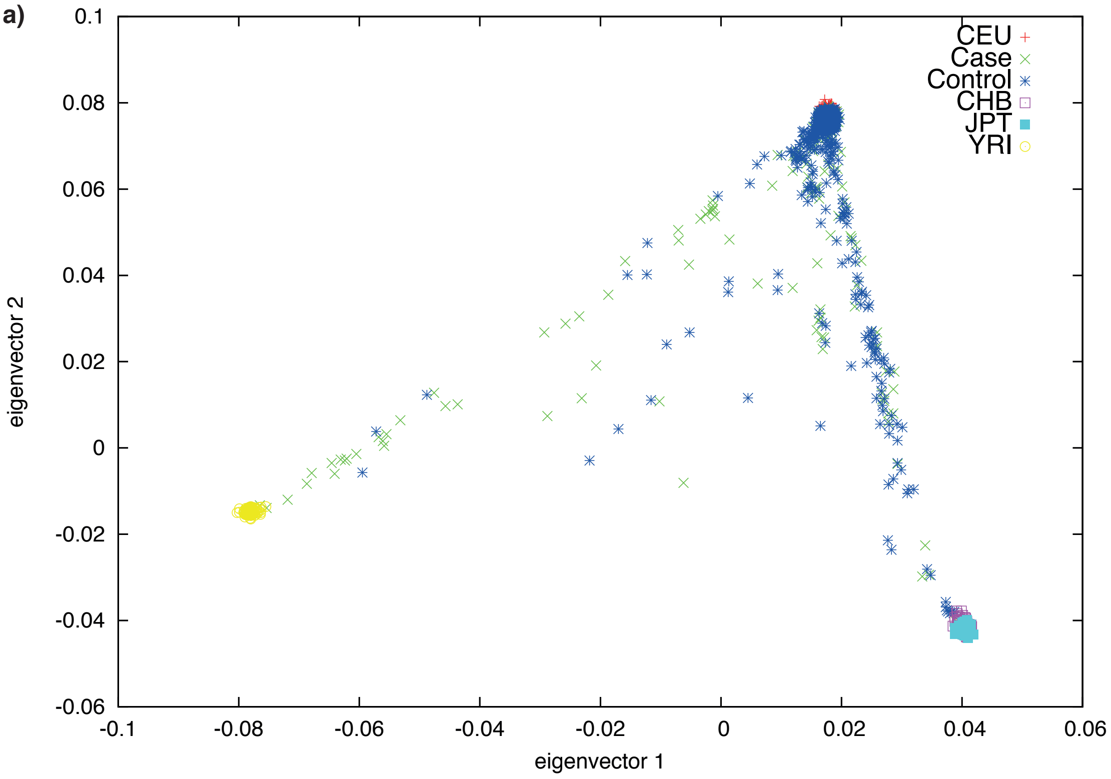

b)

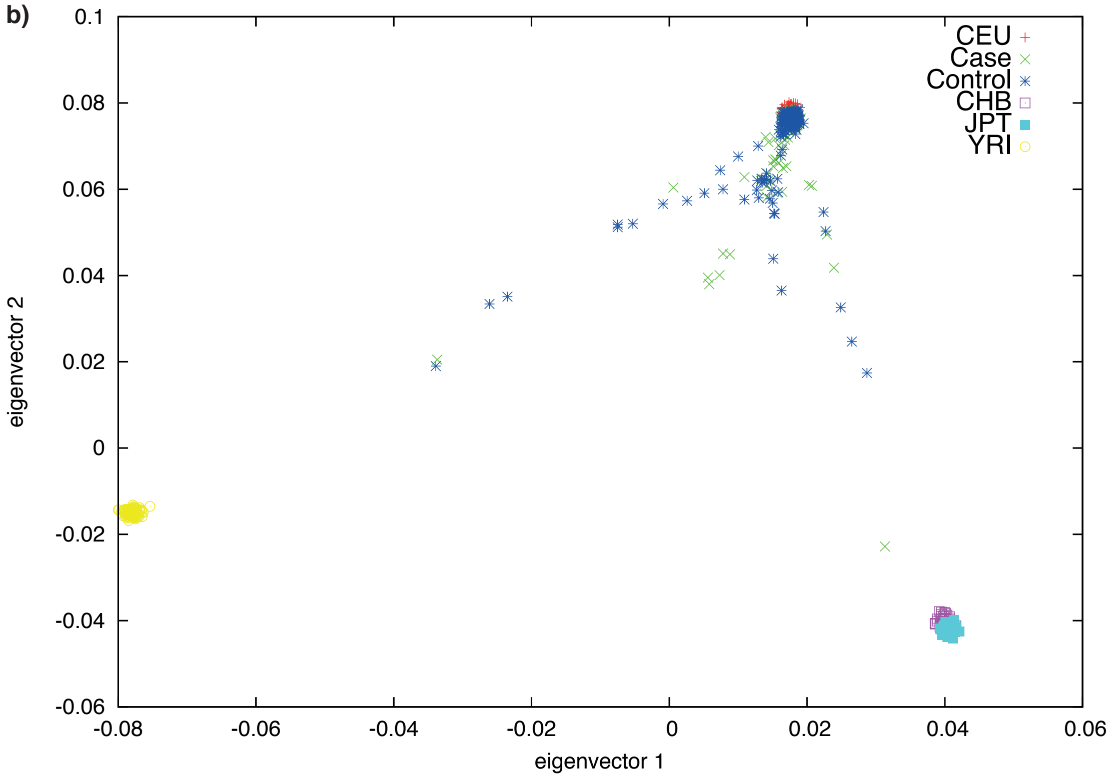

Supplementary figure 25: Projection of samples onto first two principal components of case (green crosses) and control (blue stars) samples from **a)** the Netherlands and **b)** Denmark with HapMap phase I populations. HapMap populations are 3 (red crosses) – CEU, European; 4 (pink squares) – CHB, Han Chinese; 5 (turquoise squares) – JPT, Japanese; 6 (yellow squares) – YRI, Yoruba Nigerians.
